# Development of bedaquiline nanoemulsions intended for paediatric multidrug-resistant tuberculosis: excipient selection and preformulation studies

**DOI:** 10.3389/fmedt.2024.1388113

**Published:** 2024-06-10

**Authors:** Taiwo Oreoluwa Ajayi, Madan Sai Poka, Bwalya Angel Witika

**Affiliations:** Department of Pharmaceutical Science, School of Pharmacy, Sefako Makgatho Health Sciences University, Pretoria, South Africa

**Keywords:** paediatric, tuberculosis, nanoemulsions (NE), drug delivery, preformulation compatibility, solubility studies

## Abstract

Preformulation investigations into the development of drug formulations, encompassing considerations related to the structure of the drug, excipients, composition, and physical attributes are crucial. This phase is pivotal in ensuring the ultimate success of nanoemulsion development. The objective of this study was to evaluate and define the properties of bedaquiline (BDQ) and the necessary excipients for the formulation of self-emulsifying BDQ-loaded nanoemulsions. To determine the saturation solubility of BDQ in various oils, an in-house validated HPLC method was used. Fourier transform infrared spectroscopy was utilised to identify and evaluate the compatibility between BDQ and the selected excipients. The water titration method was used to construct phase diagrams to identify the type of structure that resulted following emulsification and to characterise the behaviour of mixtures along dilution paths. The solubility studies revealed that BDQ exhibited the highest solubility in olive oil, with a solubility of 3.45 ± 0.041 mg/ml. The design space led to the formation of emulsions categorised as Winsor products. Importantly, the FTIR data indicated the absence of any potential interactions between BDQ and the chosen excipients. The preformulation studies were successful and facilitated the selection of compatible and suitable excipients for the formulation of BDQ-loaded nanoemulsions.

## Introduction

Preformulation studies are a set of experiments that focus on understanding the physicochemical properties of a drug candidate and excipients that could affect the drug performance and the development of a dosage form (1, 2). Preformulation studies can accurately predict the difficulties that will be encountered when combining the active pharmaceutical ingredient (API) with suitable excipients that will deliver a therapeutic agent to a patient in a safe, predictable and efficient manner (3). To design and evaluate the final dosage form, knowledge of physicochemical properties can be used to improve drug solubility, dissolution, permeability, and stability, as well as to choose suitable excipients and processing conditions (4).

Preformulation studies also guide formulation development with regard to drug structure, excipients composition and physical structure (5). The decision to formulate a successful drug candidate is not solely based on pharmacological effectiveness. In practice, physicochemical properties such as solubility, stability, pH, pK_a_, and log P etc. of a drug compound influence how it will be processed during formulation as well as the resultant formulation stability (6). Preformulation studies further provide cardinal information regarding API-excipients interactions which ultimately affect the bioavailability of the API (7) Several attributes of the API are characterised during the preformulation stage, including solubility, stability (solid-state and solution-state), permeability, dissolution, polymorph/salt screening, ionisation properties, particle size distribution, API-excipient compatibilities etc (8).

Nanoemulsions are thermodynamically stable colloidal dispersion systems made up of two immiscible liquids combined with emulsifying agents (surfactants and co-surfactants) to produce a single phase. There has been a significant amount of research conducted on nanoemulsions as modes of drug delivery (9). Nanoemulsions have globule diameters typically in the range of 20–600 nm though some scientific literature has proposed an upper limit for particle size in the range of 300–1,000 nm (10, 11). These systems appear optically clear and exhibit improved stability against droplet flocculation and coalescence. In addition, nanoemulsions demonstrate the potential for efficient oral, parenteral, ophthalmic, and topical systemic distribution of active compounds, such as food ingredients and lipophilic drugs (12).

**Figure 1:**
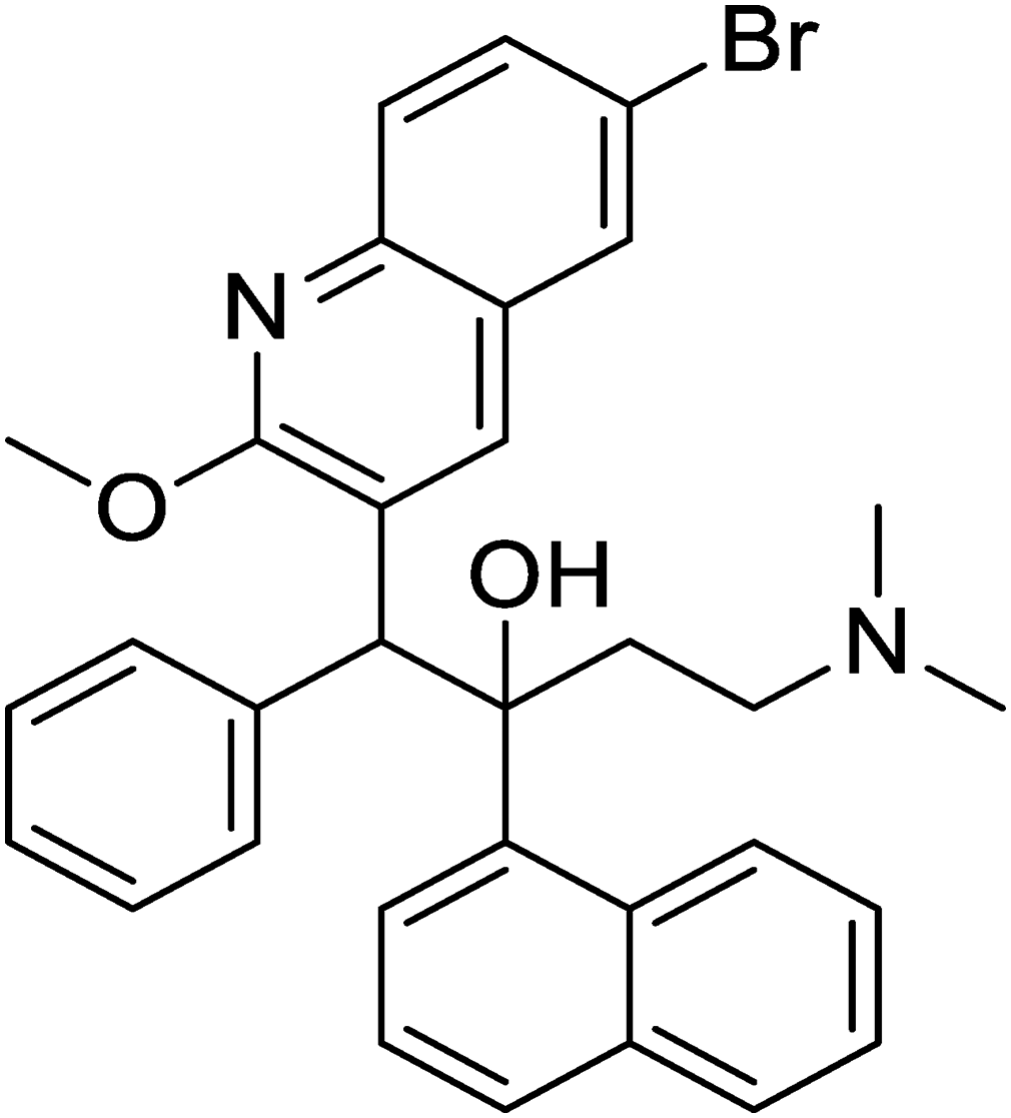
Molecular structure of bedaquiline.

Bedaquiline (BDQ) is an inhibitor of mycobacterial adenosine triphosphate, for the treatment of pulmonary multi-drug resistant tuberculosis as part of combination therapy when an effective treatment regimen is otherwise unavailable (13). It was approved by both the Food Drug Administration (FDA) and European Medicines Agency (EMEA) in December 2012 (14). In various countries, BDQ has been approved as a 100 mg tablet and more recently as a 20 mg dispersible tablet formulation (15). This formulation allows for daily administration over two weeks, followed by three times weekly dosing for 24 weeks (16). However, there is need to develop a novel drug release formulation tailored for paediatric use to overcome the constraints of the current dosage form. BDQ, as an active pharmaceutical ingredient, presents the challenge of being insoluble in aqueous media (17). The high logP value of 7.71 for BDQ indicates its lipophilic nature, making it a promising candidate for incorporation into a lipid-based drug delivery system. The chemical structure of Bedaquiline is depicted in Figure 1.

In the formulation of stable nanoemulsions, several performance indices, such as optimal Hydrophilic-Lipophilic Balance (HLB), detergency, viscosity, and Krafft or cloud point, can be used as guidelines when selecting a surfactant system (18). Non-ionic surfactants such as Spans® and Tweens® are used as emulsifying agents to create stable emulsion systems for a variety of applications. They are frequently combined in different ratios to achieve the ideal emulsification stability to target various kinds of oil-water systems (19).

Herein preformulation studies were conducted to assess the physicochemical characteristics of BDQ and analyse excipients required for the preparation of the self-emulsifying BDQ-loaded nanoemulsions. The choice of suitable oil, surfactant and co-surfactant for the formulation process was determined by the outcomes of solubility studies performed with BDQ, utilising a variety of vegetable oils and surfactants.

## Materials and methods

### Materials

BDQ was purchased from Iffect Chemphar Co. Ltd (Hong Kong, China). Tween® 80, Tween® 20, Span® 80 and Span® 20 were donated by BASF (Johannesburg, Gauteng, South Africa). Ethanol was purchased from Fisher Scientific (Massachusetts, United States). Sunflower oil, olive oil, flaxseed oil, peanut oil, corn oil and soybean oil were purchased from Escentia Products (Johannesburg, Gauteng, South Africa).

## HPLC quantification of BDQ

In this work, an ultra-fast liquid chromatographic (UFLC) system from Shimadzu equipped with a SIL-20AC auto-sampler, an SPD-M20A photodiode array detector, and an LC-20AD solvent delivery module (Kyoto, Japan) was utilised. Data acquisition, processing and reporting were achieved using Shimadzu LabSolutions CS 6.81 software (Kyoto, Japan). The mobile phase consisted of ammonium acetate buffer and methanol at a 10:90 ratio, adjusted to pH 5.4 using 1.03 ml of glacial acetic acid. Both methanol and buffer solution were degassed by filtering through a 0.45-µm HVLP Millipore® filter membrane. The detector was operated at 226 nm with the injection volume fixed at 10 µl. The HPLC analysis was conducted at 25°C.

### Screening of excipients

#### Solubility of BDQ in vegetable oils

The solubility of BDQ in different vegetable oils was determined by adding an excess amount of BDQ to 5 ml of each oil in polytope vials with caps. The tubes were agitated with the aid of cylindrical, length 20 mm, diameter 8-mm magnetic stirrer bars at 100 rpm for 48 h at ambient temperature using a magnetic stirring hot plate (Lasec®, Cape Town, South Africa).

The samples were removed and centrifuged using a centrifuge at 3,000 rpm for 15 min after which a 500 µl aliquot of the supernatant was collected and made up to 50 ml using 10:90 ammonium acetate buffer and methanol in a volumetric flask. This was then sonicated in an ultrasonic bath (Scientech®, Midrand, South Africa). The solution was filtered with a Nylon Syringe Filter 25 mm, 0.45 µm (Scharlab®, Barcelona, Spain). The concentration of BDQ in the oils was determined using an in-house developed and validated HPLC method. The experiments were conducted in triplicates.

#### Selection of surfactants

A screening process was conducted using a series of non-ionic surfactants, including Tween® 80, Tween® 20, Span® 80, and Span® 20. Tween® 80 and Tween® 20 were specifically weighed and placed into a set of CC Imelman® polytope vials (Johannesburg, Gauteng, South Africa). Olive oil was then added to these test tubes in a 1:9 (oil: surfactant) w/w ratio, to obtain 1 g per batch. Subsequently, the components were thoroughly mixed and agitated using a Fisherbrand™ ZX3 vortex mixer (Fisher Scientific, Massachusetts, United States of America). Following this, 1 ml of 20% (v/v) ethanolic solution was introduced into each oil-surfactant mixture using a micropipette. After the addition, the mixture was vortexed for 60 s at ambient temperature. Visual observations were made, and the clarity or turbidity of each sample was noted. The surfactant that resulted in a clear system was selected.

### Characterisation of pilot nanoemulsions

#### Droplet size and polydispersity index (PDI)

The PDI and droplet size of the pseudo-binary mixture was determined using a Nanotrac wave II zetasizer (Microtrac, Osaka, Japan). The sample was prepared by diluting the mixture in distilled water (1:100) and placed into a 355 × 381 × 330 mm cuvette. It was analysed at a scattering angle of 180°C at 25° and light scattering data was analysed by backscattered laser-amplified scattering reference method. The experiments were conducted in triplicates.

#### Zeta potential

The Zeta Potential (ZP) of each dispersion was determined using a Nanotrac wave II zetasizer (Microtrac, Osaka, Japan). The sample was prepared by diluting the mixture in distilled water (1:100) and placed into a 355 × 381 × 330 mm cuvette. It was analysed at a scattering angle of 180°C at 25° and light scattering data analysed by backscattered laser-amplified scattering reference method. The experiments were conducted in triplicates.

#### Phase behaviour and pseudo-ternary phase diagram construction

Tween® 80, Span® 20, and ethanol surfactant solutions were combined in the following respective ratios 1.5:0.5:1 and 1:0.5:1(w/w) using a Fisherbrand™ ZX3 vortex mixer (Fisher Scientific, Massachusetts, United States of America) at 800 rpm for 60 ± 2 s to form a surfactant mixture (S_mix_). In this method specific weights of the surfactant and cosurfactant (S_mix_) were mixed with different weights of the oil in ratios from 1:9, 2:8, 3:7, 4:6, 5:5, 6:4, 7:3, 8:2, 9:1 (w/w) to make a total of 5 g. Each of the tested ratios corresponds to a ternary phase diagram dilution line from one to nine.

After the addition of a predetermined volume of water, between 5% and 95% of the total formulation volume, the mixture was thoroughly mixed after an aliquot of water and was left to equilibrate for 24 h. The formulations were divided into 4 categories based on visual observation viz., a clear gel, a clear nanoemulsion, a milky emulsion, and a milky emulgel. The titration chart and Gibb's triangle plots were developed using a Ternaryplot software 2023 (Jules Blom, Netherlands).

#### Fourier transform infrared spectroscopy (FTIR)

The investigation of potential chemical interactions between BDQ, excipients and mixtures of thereof was investigated using FTIR. The infrared (IR) absorption spectrum of BDQ and 1:1 mixture of BDQ and olive oil, and a ternary mix of BDQ, olive oil, S_mix_ (Span® 20 and Tween® 80, ethanol) were generated using a Cary 630 FTIR spectrometer (Agilent Technologies® Inc, Santa Clara, United States of America) over the wave number range 500–4,000 cm^−1^ at a spectral resolution of 2 cm^−1^.

The mixtures and samples were mounted onto a diamond crystal and the spectral data was collected and subsequently were processed using MicroLab FTIR Software Cary 630 (Agilent Technologies® Inc, Santa Clara, United States of America). The experiments were conducted in triplicates.

## Results

### HPLC quantification of BDQ

The HPLC method developed herein resulted in the elution of bedaquiline at a retention time of 7.5 min. The method was linear in the concentration range of 0.5–300 µg/ml with LOD values of 0.039 μg/ml and LOQ of 0.12 μg/ml.

### Selection of lipids

#### Solubility of oils

The highest solubility of BDQ was observed in olive oil with a corresponding saturation solubility of 3.54 ± 0.04 mg/ml and was, therefore, the most ideal oil phase for the construction of pseudo-ternary phase diagrams for a self-emulsifying nanoemulsion.

Olive oil is mostly composed of monounsaturated fats, with oleic acid being the most prevalent fatty acid. Monounsaturated fats have a longer hydrocarbon chain, which could promote the solubilisation of BDQ, a highly lipophilic drug (20). Olive oil also contains less polyunsaturated fat than sunflower, corn, and flaxseed oil. Polyunsaturated fats can be more susceptible to oxidation, limiting their stability and ability to solubilise APIs (21). The results of solubility studies are summarised in Table 1.

**Table 1:** Saturation solubility of BDQ in vegetable oils.

Oil type	Mean Bedaquiline solubility mg/ml
Sunflower oil	2.75 ± 0.29***
Olive oil	3.54 ± 0.04
Peanut oil	2.51 ± 0.29***
Soybean oil	3.48 ± 0.33*
Corn oil	1.51 ± 0.35***
Flaxseed	3.19 ± 0.18***

Shows significant differences to olive oil in solubility with “*” indicating *p* < 0.05, “**” indicating *p* < 0.01 and “***” indicating *p* < 0.0001.

### Selection of surfactant

Tween® 80 showed a high solubilisation capacity of olive oil compared with Tween® 20. The HLB value of the Tween surfactants indicates its affinity for water (hydrophilicity) and oil (lipophilicity). When Tween® 80 is mixed with water-oil mixture, the hydrophilic heads interact with the water molecules while the lipophilic tails remain in contact with the olive oil. The dual nature of Tween® 80 allows it to bridge the gap between the hydrophilic aqueous phase and the lipophilic olive oil, emulsifying and solubilising the oil in the aqueous environment. Tween® 80 can form stable emulsions with olive oil by surrounding and solubilising the oil droplets in the aqueous phase, resulting in oil-in-water emulsions. Tween® 80 tail group is composed of unsaturated oleic acid, which is structurally similar to the tail group in olive oil. Tween® 80 tail group comprises a long chain (C_18_) of unsaturated oleic acid, while Tween® 20 is structurally composed of a medium-chain carbon tail (C_12_, lauric acid). The structural similarity between the lipophilic tails of Tween® 80 and olive oil can promote solubilisation capacity. The results for the solubilisation capacity of blends of surfactants showed that Tween® 80/Span® 20 has the highest solubilisation capacity compared with the Tween® 80/Span® 80 blend as it produced a clearer system. Surfactants that form W/O nanoemulsion have an HLB range of 3–8, whereas those that form O/W nanoemulsion such as Tween® 80 have an HLB range of 8–18. Since they are less sensitive to pH and ionic changes, non-ionic surfactants are typically thought to be better suited for pharmaceutical formulations as they exhibit lower tissue irritancy and are generally safer (22).

### Pseudo-ternary phase diagram construction

The water titration method was used to generate phase diagrams, characterise the behaviour of mixtures along dilution paths and identify the kinds of emulsions that are produced after emulsification (23). The emulsions produced by the 9:1 pseudo-binary solution surfactant to oil ratio had the lowest polydispersity index (PDI) and smallest droplet sizes (DS) of all the ratios examined as depicted in Table 2.

**Table 2:** Investigation of droplet size, polydispersity index and zeta potential for different surfactant-to-oil ratio.

Sample S_mix_: oil	Droplet size nm	PDI	Zeta potential mV
9:1	293.8	0.1233	20.1
8:2	330	0.1405	4.6
7:3	347	0.1422	11.5
6:4	478	0.2717	14.2
1:1	558	0.2980	12.0
4:6	571	0.603	8.1
3:7	717	0.967	7.8
2:8	748	0.895	6.4
1:9	1,191	1.036	3.4

Tween® 80, Span® 20, and ethanol surfactant solutions were combined in the following respective ratios 1.5:0.5:1 and 1:0.5:1 (w/w)*.* The area of the nanoemulsion increased with an increase in Tween® 80 content in the surfactant mixture. The Winsor IV regions and the gel region of the o/w emulsion exhibited an increased area with a decrease in Tween® 80 content. Tween® 80 possesses both hydrophilic and lipophilic moieties in its molecular structure. This amphiphilic nature allows it to reduce the interfacial tension between immiscible liquids, promoting the formation of small droplets during the emulsification process (24). As a result, it plays a key role in breaking down larger oil droplets into nanometre-sized ones, leading to the creation of a stable nanoemulsion.

**Figure 2:**
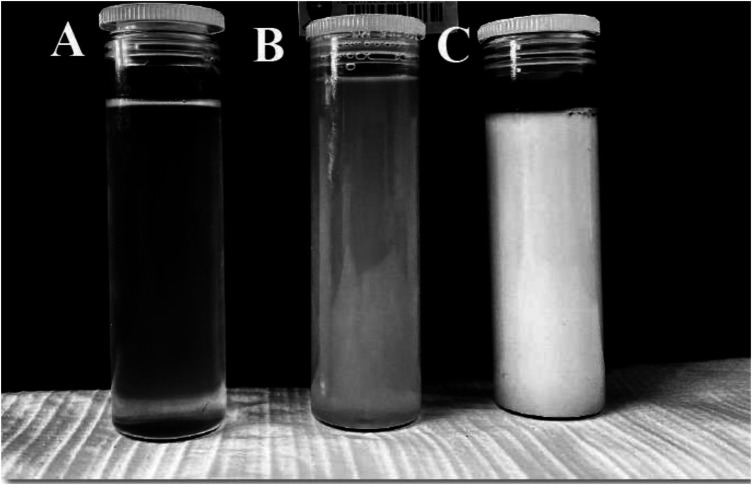
Depiction of the physical appearance of (**A**) nanoemulsion (**B**) winsor translucent (**C**) winsor milky.

It was observed that the area of the nanoemulsion decreased as ethanol content of the surfactant mixture increased, the milky isotropic and two or three-phase regions of the o/w emulsion exhibited an increased area whereas the gel region decreased in size with an increase in ethanol content. This occurred because the flexibility of the interface film led to the disturbance of the solid structure as the fluid phase area expanded (25). The physical appearance of a nanoemulsion and Winsor milky and translucent mixtures are depicted in Figure 2.

**Figure 3:**
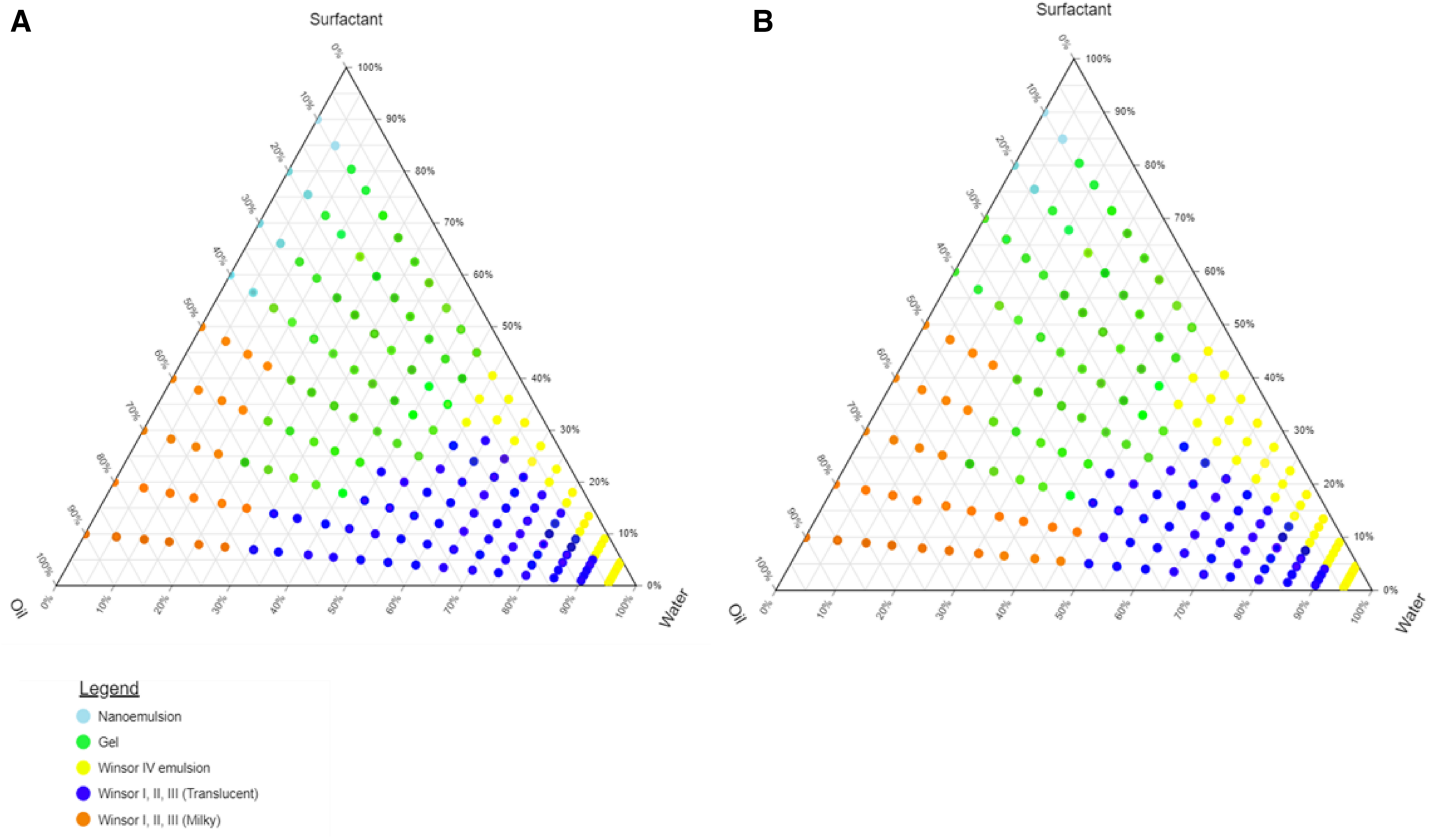
Pseudo-ternary phase diagram of olive oil with surfactant-mixture (**A**) Tween 80®, ethanol, Span 20® (1.5:0.5:1) and (**B**) Tween® 80, ethanol, Span® 20 (1:0.5:1).

The different regions that were formed by the 9:1 surfactant-to-oil mix in different surfactant solutions Tween® 80, Span® 20, and ethanol surfactant combinations in the ratio 1.5:0.5:1 and 1:0.5:1 (w/w) are depicted in Figure 3.

### Characterisation of selected excipients

#### Fourier transform infrared spectroscopy

**Figure 4:**
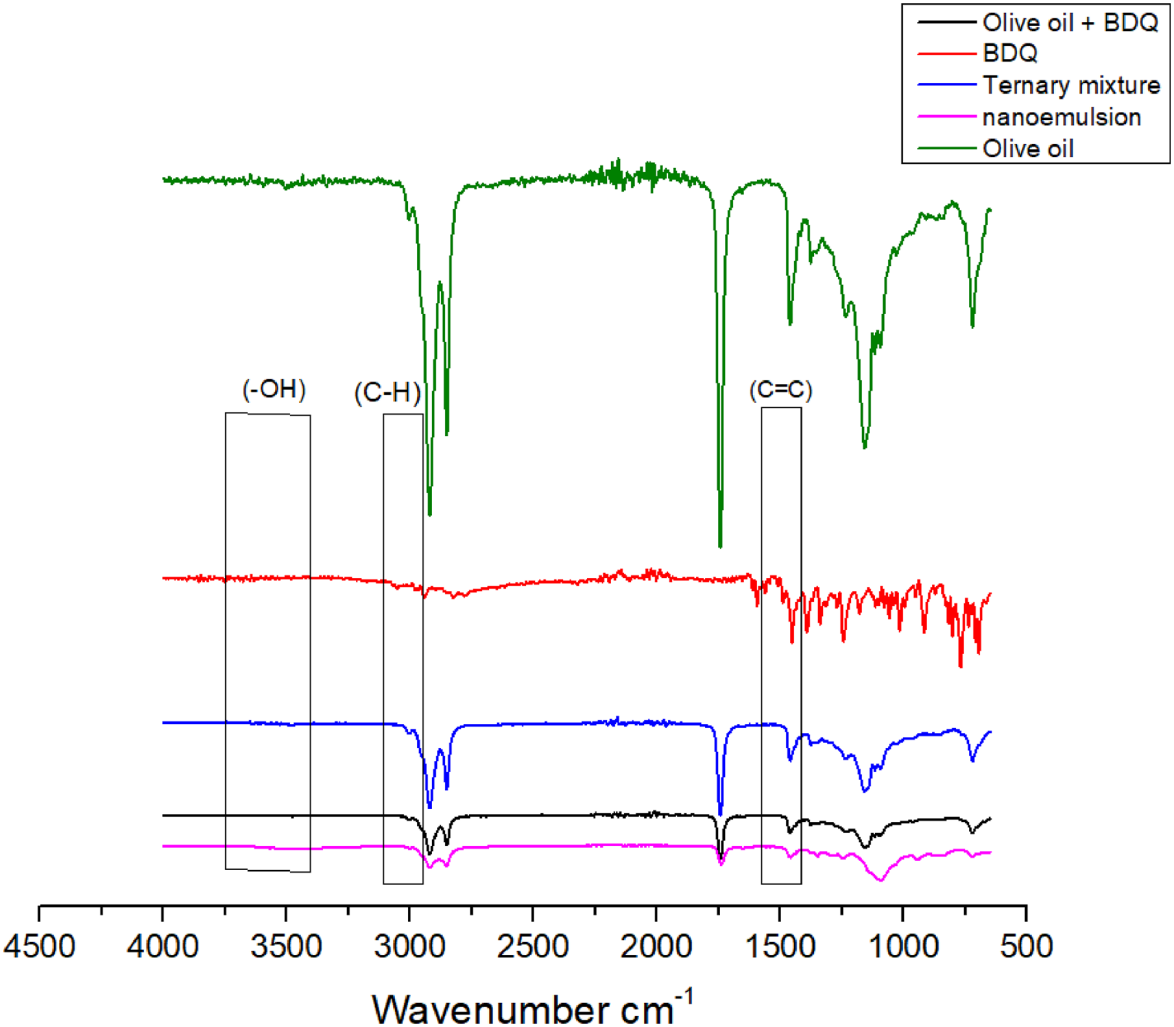
FTIR spectra of BDQ, 1:1 mixture of BDQ and olive oil, ternary mixture of 1:1:1 excipient (Tween® 80, Span® 20, ethanol) and nanoemulsion.

The IR spectrum enables the detection of specific unsaturated bonds, aromatic rings, and functional groups in molecules, as well as their orientation and position (26). The spectrum for BDQ and binary mixtures of BDQ and different is depicted in Figure 3 and the spectrum of a ternary mix of BDQ, olive oil, and Tween® 80, Span® 20 and ethanol (S_mix_) is presented in Figure 4.

FTIR spectra of 1:1 binary mixture of BDQ in vegetable oils revealed the presence of the characteristic peaks of BDQ in the same spectral regions for the -OH group (3,083 cm^−1^) and aromatic (2,960, 2,956 cm^−1^ and 1,631 cm^−1^). It is also characterised by prominent absorption peaks at 1,546 cm^−1^ (C = C stretch in aromatic ring) The intensity of these peaks were diminished probably due to encapsulation of BDQ and a consequence of reduced concentration due to dilution (27).

Vegetable oils exhibit comparable functional groups, despite having distinct triglyceride compositions. Consequently, distinguishing variations in the oil compositions within this category using FTIR is challenging.

There are no observable interactions between BDQ and olive oil, and all spectra display the presence of identical functional groups. In the ternary mixture, the characteristic molecular peaks associated with known and anticipated functional groups of BDQ are clearly visible. Importantly there is no emergence of new peaks or any unexpected functional groups. This absence of such spectral changes signifies that no chemical interactions took place between BDQ and the olive oil. This observation suggests that it is feasible to produce stable formulations with these components.

## Conclusion

Selection of oils, surfactants, co-surfactants, and constructing phase diagrams are essential prerequisites for formulating drugs into nanoemulsions. The choice of suitable components such as oil, surfactant, and co-surfactant for the formulation process was determined based on the outcomes of solubility experiments conducted with BDQ using various vegetable oils and surfactants. Among the tested oils, olive oil, exhibited the highest solubilisation potential of BDQ and was consequently selected as the vegetable oil component for BDQ-loaded nanoemulsion and was attributable to the triglyceride composition.

Studying the phase behaviour of component mixtures is valuable for optimising formulations, and pre-formulation studies help determine the suitable proportions for each component. Additionally, they aid in decision-making regarding manufacturing processes. The purpose of these phase diagrams was to ensure that the formulations could readily form nanoemulsions by determining the appropriate ratios of water, oil and S_mix_ components. The S_mix_ ratio 1.5:0.5:1 was used for the formulation as it produced the highest region of nanoemulsion of the ternary phase diagram.

Thorough preformulation investigations are essential to identify suitable excipients for inclusion in self-nanoemulsifying drug delivery systems, ensuring the selection of excipients that optimise product performance. The preformulation studies conducted herein showed that a stable nanoemulsion can be formulated and forms the basis for the development and optimisation of a nanoemulsion capable of safely delivering BDQ to paediatric patients.

While the success of these studies provides a basis for nanoemulsion development for paediatrics, optimisation studies are required to minimise the ethanol content for paediatric formulations while providing a nanoemulsion with suitable critical quality attributes viz droplet size, zeta potential and polydispersity index which will likely translate to a formulation meeting ideal quality target product profiles (long term stability, good organoleptic properties, high efficacy etc).

## Data Availability

The original contributions presented in the study are included in the article/Supplementary Material, further inquiries can be directed to the corresponding authors.
